# Dissolution Rate Enhancement of Clarithromycin Using Ternary Ground Mixtures: Nanocrystal Formation 

**Published:** 2013

**Authors:** Malihe Shahbaziniaz, Seyed Mohsen Foroutan, Noushin Bolourchian

**Affiliations:** a *Department of Pharmaceutics, School of Pharmacy, Shahid Beheshti University of Medical Sciences, Tehran, Iran.*; b *Student Research Committee, School of Pharmacy, Shahid Beheshti University of Medical Sciences, Tehran, Iran.*

**Keywords:** Clarithromycin, Nanocrystals, Co-ground mixture, Dissolution rate

## Abstract

Clarithromycin (CLA), a broad-spectrum macrolide, is a poorly soluble drug with dissolution rate limited absorption. The aim of this investigation was to prepare CLA nanoparticles from a ternary ground mixture in the presence of sodium lauryl sulfate (SLS) and polyvinyl pyrrolidone (PVP) as co-grinding water-soluble compounds, in order to improve the drug dissolution rate. Different weight ratios of CLA: SLS: PVP were ground in a dry process by planetary ball mill using different grinding ball size. Following the dissolution rate study, physical properties of the best dissolved co-ground formulation was studied. The accelerated stability studies were also conducted on the co-ground formulation.

The results revealed that the dissolution rate of ternary ground mixtures was much higher than that of the intact drug (p < 0.001). Decreasing the grinding ball size and weight with the same rotation speed resulted in particles with decreased dissolution. On the other hand, increasing the PVP concentration in the formulations reduced the drug dissolution. Dissolution efficiencies (DE_10_ and DE_30_) for the best dissolved formulation, which consisted of the equal ratio of each co-ground component, were 8.7 and 5 folds higher than the untreated CLA, respectively. This formulation formed nanocrystals with enhanced solubility after dispersing in water. X-ray diffraction, differential scanning calorimetry and infrared spectrophotometry confirmed no chemical interaction and phase transition during the process. Accelerated stability studies confirmed that the co-ground mixture almost remained unchanged in terms of dissolution rate, drug assay and particle size after exposing in stability conditions for three months.

## Introduction

Aqueous solubility of drugs is one of the important properties in the achievement of desirable formulation. Many active substances are poorly soluble or insoluble compounds which show reduced dissolution rate and bioavailability as well as erratic absorption after oral administration. Since many years, various formulation approaches were used to overcome this problem, such as salt formation, using solubilizing agents and drug carriers, which are not applicable for all poorly soluble drugs (1).

Decreasing the particle size, micronisation, is a classical method of dissolution rate enhancement due to the increased surface: volume ratio and subsequently improved drug absorption. In fact, micronisation is a simple technology especially for class II drugs of biopharmaceutical classification system (BCS), having good permeability but poor solubility and low dissolution rate. However, for very poor soluble drugs, the dissolution rate enhancement by micronisation is not sufficient to lead to a high bioavailability. Therefore, nanonization is considered as a promising approach in which drug nanocrystals with the mean particle size below 1 μm are prepared (2). Enhancement of the saturated solubility of drug nanocrystals as well as the effective surface area in contact with the dissolution medium, directly could increase the drug dissolution rate and therefore improve the oral bioavailability (3, 4).

Among different methods of nanocrystals preparation, mechanical comminuting (grinding) is an effective and simple technique by which large quantities of the modified material could be obtained (5). Grinding can be operated in wet or dry media. Different materials were prepared successfully as nanosuspensions by wet grinding method (6-8). The wet method was shown to be more efficient in producing particles with reduced size rather than dry grinding method (9). However when a dry product is needed, drying step following the wet milling could be time and money consuming. In addition, the wet technique could not be applied for drugs that are susceptible to hydrolytic degradation. In those conditions dry grinding method might be applied effectively with no need to any aqueous or organic solvents. Co-grinding of poorly soluble drugs with water soluble additives (polymers and surfactants) was shown to be an effective method in producing nanosized particles with enhanced solubility (10-16). Co-ground mixture of nifedipine with polyethylene glycols and hydroxypropyl methylcellulose showed enhanced solubility compared to the intact drug following nanoparticles formation (17). Furthermore, nanosized particles with crystalline properties could be obtained by co-grinding method (18). With regard to the instability of amorphous particles, it could be considered as an advantage.

Clarithromycin (CLA), a semi-synthetic macrolide, is a broad-spectrum antibiotic with excellent activity against gram positive, some gram negative and anaerobic bacteria (19). CLA is used in the treatment of a number of bacterial diseases such as gastric *Helicobacter pylori *infection (20) as well as upper respiratory, skin and otolaryngology infections (21). It has a biological half life of about 3-5 h (22) and undergoes first-pass metabolism after oral administration (23). CLA belongs to the class II of BCS having low solubility which resulted in a dissolution rate-limited absorption and low bioavailability (24). 

Few approaches have been reported for dissolution rate enhancement of CLA. Complexation with cyclodextrin was one of the methods that could enhance CLA solubility to a great extent, but higher stability of the complex probably resulted in slightly lower efficacy against *Mycobacterium avium *compared to the free drug (25). Inoue et al. used ascorbic acid 2-glucoside as solubilizing agent and increased the CLA solubility by co-grinding method (26). An amorphous modification of CLA by grinding and spray drying was also studied by Yonemochi *et al. *(27). 

The purpose of this study was to improve the dissolution rate of CLA by nanoparticles formation using ternary dry ground mixtures and physicochemical evaluation of the resulted nanocrystals .

## Experimental

Clarithromycin was supplied from Shifa Pharmed Co., (Iran). Polyvinyl pyrrolidone (PVP K10) and sodium lauryl sulfate (SLS) were obtained from Sigma Chemical Co. and Merck (Germany), respectively. All other chemicals and solvents used were of pharmaceutical grades.

*Preparation of ternary ground mixtures*

Planetary ball mill (PM100, Retsch Co., Germany) was used for grinding of particles. A 50 ml grinding bowl and balls, all of zirconium oxide, were used for sample preparation. Based on our preliminary experiments (unpublished data), the rotation speed and time of grinding were fixed at 400 rpm and 60 min, respectively. The weight of grinding sample was kept constant in different formulations.

In order to investigate the effect of size and number of grinding balls on particles dissolution, two series of grinding balls with similar surface area were used as follows:

A: 5 balls with 10 mm diameter in combination with 20 balls with 5 mm diameter

B: 10 balls with 10 mm diameter

In the present investigation, SLS and PVP were used as co-grinding components with different drug: surfactant: polymer weight ratios (Table 1). 2.5 g of physical mixtures were loaded into the bowl and dry milled. The compositions of different preparations are summarized in Table 1.

**Table 1:** The composition of CLA ternary ground mixtures, grinding balls and dissolution efficiencies calculated for each sample

**Formulation no.**	**Drug : SLS : PVP ratio**	**Ball number and type ** ^a^	**DE**_10_ **(%) (n=3)**	**DE**_30_ **(%) (n=3)**
CLA ^b^	1 : 0 : 0	-	8.26 ± 1.52	17.81 ± 1.35
S1	1 : 0.2 : 1	A	46.98 ± 0.36	60.36 ± 0.10
S2	1 : 0.2 : 5	A	36.49 ± 0.25	53.90 ± 0.71
S3	1 : 0.2 : 1	B	52.41 ± 0.21	67.80 ± 0.46
S4	1 : 0.6 : 1	B	62.38 ± 6.15	89.88 ± 2.48
S5	1 : 1 : 1	A	68.72 ± 1.04	84.10 ± 0.78
S6	1 : 1 : 1	B	72.18 ± 1.02	88.85 ± 0.85
S7	1 : 1 : 3	A	53.17 ± 3.38	77.91 ± 3.37
S8	1 : 1 : 3	B	61.70 ± 0.35	83.87 ± 1.80
S9	1 : 1 : 5	B	47.04 ± 0.41	67.65 ± 0.13
PM c	1 **: **1 : 1	-	41.57 ± 2.55	57.25 ± 3.27

^a^ A: 5 balls with 10 mm diameter in combination with 20 balls with 5 mm diameter, B: 10 balls with 10 mm diameter

^b^ CLA: The intact drug

^c ^PM: Physical mixture (unmilled)

*Preparation of physical mixtures*

Physical mixtures of different ratio of CLA, SLS and PVP were prepared by mixing them for 5 min using vortex mixture in a glass bottle.

*Dissolution studies*

The dissolution test was performed using a USP no. II rotating paddle apparatus at 37oC and a rotating speed of 50 rpm in 500 mL of phosphate buffer solution (pH 7.4). Equivalent to 25 mg CLA of the prepared co-ground samples or the intact drug were dispersed in the dissolution medium. Samples (1 mL) were withdrawn at predetermined time intervals and replaced with the same volume of fresh media after each withdrawal. Samples were centrifuged at 10,000 rpm and 10 min for three times. After filtration, the amount of drug in supernatant was determined using HPLC method described below. Each formulation was analyzed in triplicate.

*Dissolution data analysis*

Dissolution efficiencies (DE_10_ and DE_30_) were calculated based on the area under the dissolution curve from 0 to 10 and 30 min, respectively, measured by trapezoidal rule, and expressed as a percentage of the area at maximum dissolution (28).

*Solubility studies*

Saturated solubility evaluations were carried out by shaking an excess amount of pure drug, ternary ground sample and related physical mixture in distilled water for 48 h at 25°C. After centrifugation, the concentration of CLA was measured by HPLC method described below. The mean results of triplicate measurements and the standard deviation were reported.

*Drug assay*

An accurately weighted sample of the co-ground mixture was dissolved in methanol to obtain a stock solution and then diluted with HPLC mobile phase to obtain a concentration of 100 μg/mL of CLA. The solution was then filtered and analyzed for drug content by HPLC method. Each sample was tested in triplicate. The percent of drug content was calculated by dividing the actual CLA content to the theoretical amount of CLA in the ground sample multiplied by 100. 

*HPLC analysis*

A validated high performance liquid chromatographic method was developed for determination of CLA in different samples. The CLA content was measured using an HPLC system (Knauer, Germany) equipped with a UV detector (Smartline 2500, Knauer, Germany) and a C18 column (Perfectsil Target ODS-3.5 μm, 250 × 4.6 mm, Analysentechnik, Germany). Mobile phase consisted of acetonitrile and potassium phosphate buffer solution (50: 50) at pH 3 was used at a constant flow rate of 0.8 mL/min and the eluent was monitored at 205 nm. 

*Particle size analysis*

Particle size of the chosen co-ground formulation was determined using a Malvern Zetasizer (Malvern Instruments, UK). Prior to the measurement, the sample was diluted with deionized water to a suitable scattering intensity and sonicated for 2 min to create a homogenous suspension.

The size of the intact drug was measured using Malvern Mastersizer 2000 (UK) based on laser diffraction method. Sample preparation method was the same as explained above. 

*Zeta potential measurement*

The surface properties of the untreated CLA and the co-ground mixture particles were examined by zeta potential (ZP) measurement using Malvern Zetasizer (Malvern Instruments, UK). Samples were prepared by the method as described for particle size analysis.

*Differential scanning calorimetry (DSC)*

The phase transition of samples was analyzed by a differential scanning calorimeter (DSC-60, Shimadzu Co., Japan) which was calibrated using indium standard. Samples were placed in sealed aluminum pans and heated up to 240 oC with the rate of 10 oC/min.

*X-ray powder diffraction (XRD)*

To evaluate the crystalline properties of prepared samples, XRD pattern was measured by a Philips Xpert diffractometer (The Netherlands) over the range of 5-40 2θ. Cukα radiation was generated at 30 mA and 40 kV with the scan rate of 1°/min.

*Infrared spectroscopy (IR)*

About 2-3 mg of the intact drug and selected samples was triturated with potassium bromide and compressed into a disc (12 mm) at 10 ton pressure. IR adsorption spectra were recorded using a spectrophotometer (Perkin-Elmer 843, UK) over a range of 200-4000 cm-1 to evaluate the molecular states of the raw material as well as prepared samples.

*Scanning electron microscopy (SEM)*

The shape and morphology of untreated CLA and co-ground sample were evaluated by SEM. Samples were mounted on a metal stub and coated with gold through a sputter-coater (Bal-tec SCD 005, Germany) before examining by a scanning electron microscope (Philips XL30, Netherlands).

*Stability studies*

In order to carry out the accelerated stability studies, the best dissolved co-ground formulation was kept at 45oC and 75 % relative humidity (RH) for 3 months after packing in suitable primary packaging. Various physical properties of the mixture including drug assay, particle size as well as drug dissolution were analyzed at the end of 3rd month.

*Statistics*

The reported data represent the mean value ± standard deviation (SD). Significance of difference was evaluated using one-way ANOVA at the probability level of 0.05.

## Results and Discussion

*Dissolution studies*

**Figure 1:**
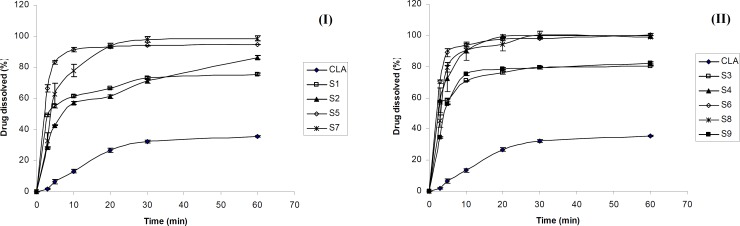
Dissolution profile of clarithromycin from the intact (CLA) and ternary ground mixtures prepared using different grinding balls according to Table (1) I: A and II: B (n=3).

Figure 1 shows the dissolution profiles of CLA as well as ternary ground samples prepared in the presence of PVP and SLS using different grinding ball numbers and sizes. 

According to Figures 1-I and 1-II, the dissolution rate of clarithromycin was very low and only 35 % of the drug was dissolved within 60 min, which was expected due to its poor solubility. However, co-ground samples reached 75-100 % of drug dissolution during the same period. Based on Table 1, dissolution efficiencies of all prepared samples were significantly higher (p < 0.001) than that of the intact drug (CLA). DE_10_ and DE_30_ values obtained for the ternary ground formulations were 4.4-8.7 and 3-5 folds higher compared to the corresponding pure drug, respectively. The increased dissolution rate of ternary ground mixtures could be attributed to two factors including particle size reduction as well as the presence of hydrophilic additives. As it is described in the XRD section, no phase transition from crystalline to amorphous state was occurred during the process. Therefore, higher dissolution rate of co-ground mixtures could not be attributed to this issue.

*The effect of grinding ball*

Based on Figure 1, grinding ball types and numbers influenced the drug dissolution rate of various samples. The dissolution rate of CLA from S3 (prepared by B type grinding balls) was higher than that of S1 (prepared with the same drug: additives ratio by A type grinding balls). The same result was observed for S6 in which the drug dissolution was slightly higher than that of S5. The difference in the dissolution profiles of S7 and S8 also could be attributed to the size and number of the grinding ball system. Table 1 shows that the DE_10_ and DE_30_ values were significantly higher for S3, S6 and S8 in comparison with S1, S5 and S7, respectively (p ≤ 0.01). Therefore, using 10 grinding balls with 10 mm diameter could be more effective in the preparation of fine particles with higher dissolution rate when compared to the combination of 10 and 5 mm diameters balls with different numbers. It seems that the weight of grinding balls is one of the factors influencing the particles properties. Grinding balls type B with the weight of 20.5 g were more effective in reducing the drug particle size and consequently dissolution rate enhancement compared to grinding balls type A with the weight of 15.8 g. It was reported that small grinding media creates lower energy during tumbling due to the lower weight that is not sufficient to break the fine particles. Increasing the rotation speed of the grinding bowl might compensate for this phenomenon (29).

*The effect of additives ratio*

Based on Figure 1-I, incorporation of higher PVP concentration in S2 compared to S1 decreased the CLA dissolution rate. In addition, for the formulations S6, S8 and S9, (all prepared by B type grinding balls and similar drug : SLS ratio), increasing the amount of PVP from 1 to 3 and 5 parts, respectively, reduced the dissolution rate (Figure 1-II) and also the values of dissolution efficiencies significantly (p ≤ 0.01) (Table 1). Considering the hydrophilicity of PVP, it was expected that increasing its concentration in the ground formulations, would improve the drug dissolution rate.The opposite achievement in the present study might be attributed to the fact that PVP is a binder which could increase the cohesion of fine particles together and causes agglomeration during grinding. The formation of agglomerated particles was confirmed by SEM which is described later. Agglomeration of particles in the presence of PVP might be intensified in higher concentration and this could be a major factor in decreasing the surface area available for dissolution. On the other hand, the higher the PVP concentration, the higher the viscosity of the diffusion layer around the particles in the medium, which may hinder the drug dissolution. Based on Vogt *et al.*, surrounding the particles with PVP may delay the drug dissolution due to a barrier formed against water penetrating (30). All these issues can be probably the cause of slower dissolution rate of samples containing higher PVP concentration especially at the early stages of dissolution test.

On the other hand, the drug dissolution rate of S4 was higher than that of S3, due to the increased SLS concentration (Figure 1-II). Referring to Table 1, significant differences was observed between DE_10_ and DE_30_ values of these co-ground mixtures (p = 0.049 and 0.0001, respectively). In fact, increasing the amount of SLS from 0.2 to 0.6 part of the formulation had a great effect in dissolution rate enhancement. Further increasing the SLS amount in the formulation S6 only improved the DE10 (p < 0.05) and did not affect the DE_30_ values. It seems that application of higher SLS concentration (as a hydrophilic surfactant) in the formulations improved the wettability of the particles and in turn the drug dissolution rate, particularly at the beginning of the test. Hence, an optimum concentration of all additives must be applied in order to obtain desirable outcomes.

Considering all prepared formulations, the best DE_10_ and DE_30_ values were obtained for S6 in which the equal concentration of PVP, SLS and CLA was used. Therefore S6 was chosen as the optimum ternary ground mixture for further studies. 

*Dissolution study of the physical mixture*

**Figure 2:**
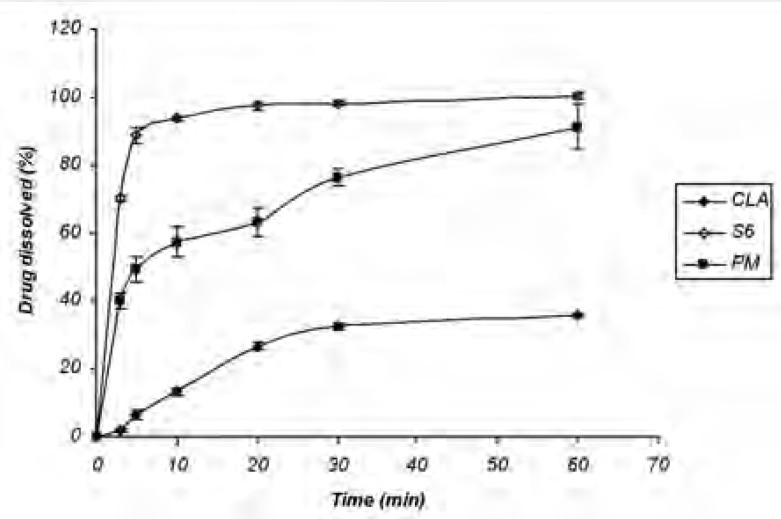
Dissolution profile of untreated drug (CLA), S6 (co-ground sample) and physical mixture (n = 3).

The increased dissolution rate of ternary ground samples could be attributed to different factors including the presence of hydrophilic additives and particle size reduction. In order to find out the effect of additives on CLA dissolution properties, physical mixture (PM) of the ingredients without grinding step was prepared and analyzed for the drug dissolution. Based on Figure 2 CLA dissolution from PM was faster than the intact drug, but slower than S6 formulation with the same composition (1: 1: 1). DE_30_ (%) values calculated for the intact drug, PM and S6 were 17.81 ± 1.35, 57.25 ± 3.27 and 88.85 ± 0.85, respectively. Therefore, CLA dissolution enhancement could be also related to the other factors rather than the presence of hydrophilic additives.

*Particle size and zeta potential measurement*

Based on the results obtained from particle size analysis (Table 2), 90 % of the intact drug particles have a size as much as 179.69 μm or less, while d (0.9) for the co-ground sample (S6) after dispersing in water was equal to 531 nm. Also, the value of d (0.5) for the prepared sample was less than 200 nm. This analysis confirmed the formation of nanoparticles by ternary ground mixtures. Therefore, nanonization of CLA could be considered as a major factor in dissolution rate enhancement.

The ZP values for the intact drug and co-ground sample (S6) were equal to −6.57 and −58.13 mV, respectively. In the other words, presence of additives in the co-ground mixture increased the ZP value significantly. Considering the anionic properties of SLS, it is probable that SLS was adsorbed onto the surface of nanoparticles. This is in accordance with the results from Itoh et al. They assumed that the particles obtained by co-ground ternary mixtures might be formed by PVP adsorption on the surface of drug nanoparticles which were in turn adsorbed by SLS (11). Typically, a colloidal solution with ZP greater than |30mV| is considered as a stable system (31). Therefore CLA nanoparticles prepared in the aqueous solution could be stable due to higher ZP value. 

*Solubility*

Based on Noyes-Whitney equation enhancement of drug saturated solubility could improve its dissolution rate (32). Table 3 shows the saturated solubility data obtained for untreated CLA, S6 (ternary ground sample) and related physical mixture (PM) in water. The drug saturated solubility of the ground sample was significantly higher than that of the intact drug (p < 0.0001). Since the solubility obtained for S6 (consisted of drug: SLS : PVP ratio of 1:1:1) was even higher (p < 0.0001) than PM, it can be concluded that the higher drug saturated solubility was not only related to the presence of water-soluble additives in the medium, but also is associated with the drug status in co-ground sample (nanosized particles). Based on XRD analysis, crystalline form of drug was not changed during the process. Therefore higher solubility of drug nanocrystals was related to the particle size reduction rather than crystalline phase transformation.

**Table 2:** Particle size of the untreated drug (CLA) and the co-ground sample (S6).

**Sample**	**Particle size ** **(μm)**		**Span ** ^a^
**CLA**	d (0.5)	68.82	
	d (0.9)	179.69	2.291
**S6**	d (0.5)	0.184	
	d (0.9)	0.531	2.76

^a ^Span: The width of the distribution

**Table 3:** Saturated solubility of CLA, S6 (co-ground sample) and PM (physical mixture) in water (n = 5, mean ± SD).

**Sample**	**CLA**	**S6**	**PM**
Solubility (μg/mL)	44.49 ± 1.619	156.22 ± 0.576	90.69 ± 0.813

*DSC analysis*

**Figure 3:**
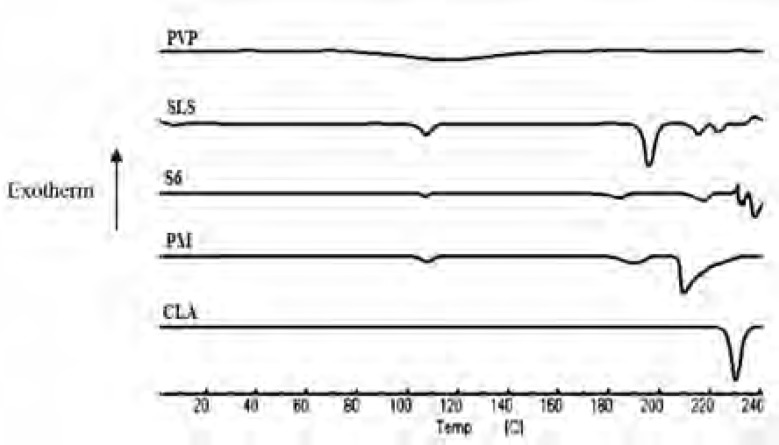
DSC thermograms for the untreated drug (CLA), PVP, SLS, co-ground sample (S6) and related physical mixture (PM).

The DSC thermograms of untreated drug (CLA), PVP, SLS, ternary ground sample (S6) and physical mixture (PM) were depicted in Figure 3. A characteristic endotherm appeared for the untreated drug at the onset temperature of 227.16°C which could be attributed to the melting of clarithromycin form II (33). A broad peak was observed in the thermogram of PVP which is related to its dehydration (34). Also, characteristic peaks were identified in the DSC curve of SLS at 107.5°C and 192.78°C (35). The DSC curve obtained for S6 (co-ground mixture) presented the same thermal profile as that of PM, suggesting no polymorphic changes during nanosizing process. It must be considered that a shift was appeared for the drug melting onset temperature to 211.57 and 207.81°C for S6 and PM, respectively, which could be due to the presence of additives on the surfaces of drug crystals in both samples (36, 37). It was shown that the presence of sodium dodecyl sulfate could influence the melting temperature of nanocrystals without alteration in crystalline properties (38). It should be noted that reduction in melting temperature could increase the drug dissolution rate. 

*Powder X-ray diffraction (XRD)*

**Figure 4:**
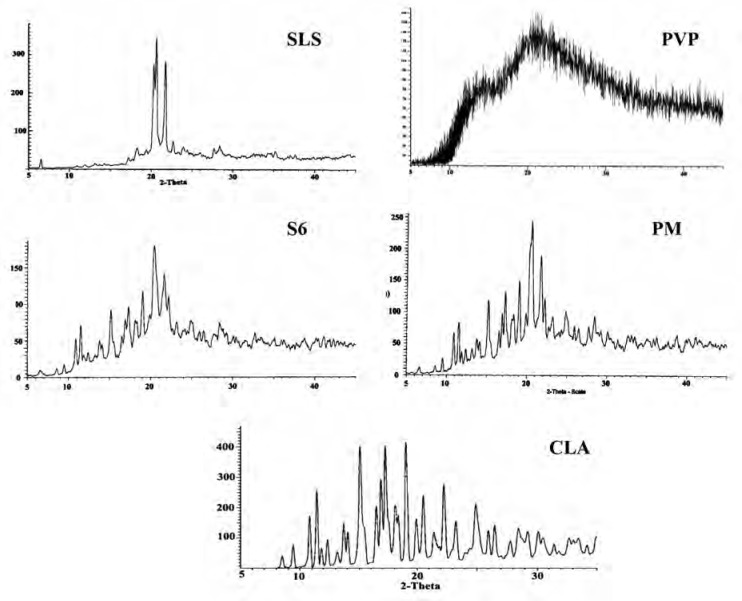
XRD patterns for the untreated drug (CLA), PVP, SLS, co-ground sample (S6) and related physical mixture (PM

The XRD patterns of CLA, PVP, SLS, ternary ground formulation (S6) and related physical mixture (PM) were depicted in Figure 4. The XRD spectra of untreated CLA exhibited characteristic peaks at diffraction angles (2θ) of 8.50, 9.44, 10.84, 11.45 and 17.25 and confirms the form II crystalline structure of the drug (19, 39) which is more thermodynamically stable than form I (33, 40). 

A broad spectrum without any distinct peak could be observed in the diffractogram of PVP (Figure 4) which confirms amorphous nature of this polymer (41), while well-defined diffraction peaks at 2θ angles of 20.31, 20.67 and 21.84 were detected for SLS (42). 

Although grinding could induce the phase transition to amorphous state, but it depends on several factors such as milling time and ball size (43). In this study, the distinct peaks of untreated drug as well as additives could be detected in ternary ground mixture and PM at the same 2θ values, indicating that crystalline state of drug was not changed following the grinding operation. The decrease in the peak intensity for nanocrystals in the co-ground mixture can be attributed to the particle size reduction in the sample (44).

*Infrared spectroscopy*

**Figure 5:**
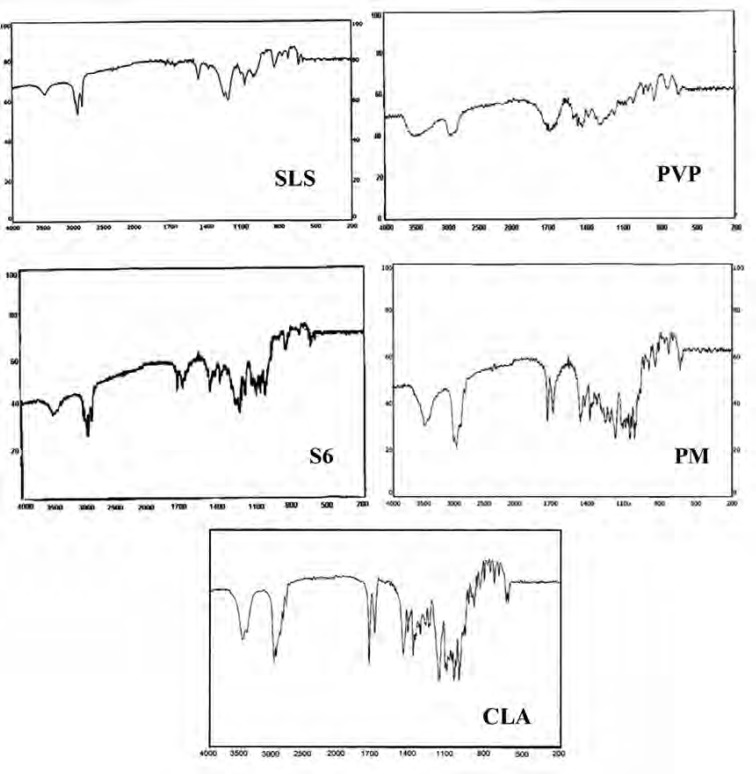
IR spectra for the untreated drug (CLA), PVP, SLS, co-ground sample (S6) and related physical mixture (PM).

IR spectroscopy was performed in order to identify any possible interaction between the drug and the additives in nanoparticles formulation. Figure 5 demonstrates the IR spectra of the intact CLA, ternary ground sample (S6) and related physical mixture (PM). Characteristic peaks of CLA were found at 1690.50 cm^-1^ (ketone carbonyl), 1729.22 cm^-1^ (lactone carbonyl), 1420 cm^-1^ (N-CH3) and 3450.15 cm^-1^ (hydrogen bonding between OH Groups) (45, 19). Also the main peaks of PVP were appeared at 1668 and 1293 cm^-1^ which were related to the C = O stretching and C-N stretching vibrations, respectively (46). SLS shows the characteristic signals at 2873 and 2955 cm^-1^ related to the C-H stretching bands and 1219 and 1249 cm^-1^ corresponding to SO2 vibrational features (47).

Both co-ground sample (S6) and PM reflected the characteristics of the components present. This indicates that the IR spectra for these samples were not associated with changes at the molecular level. In fact, no interaction seems to be occurred during co-grinding process. 

*Scanning electron microscopy*

**Figure 6:**
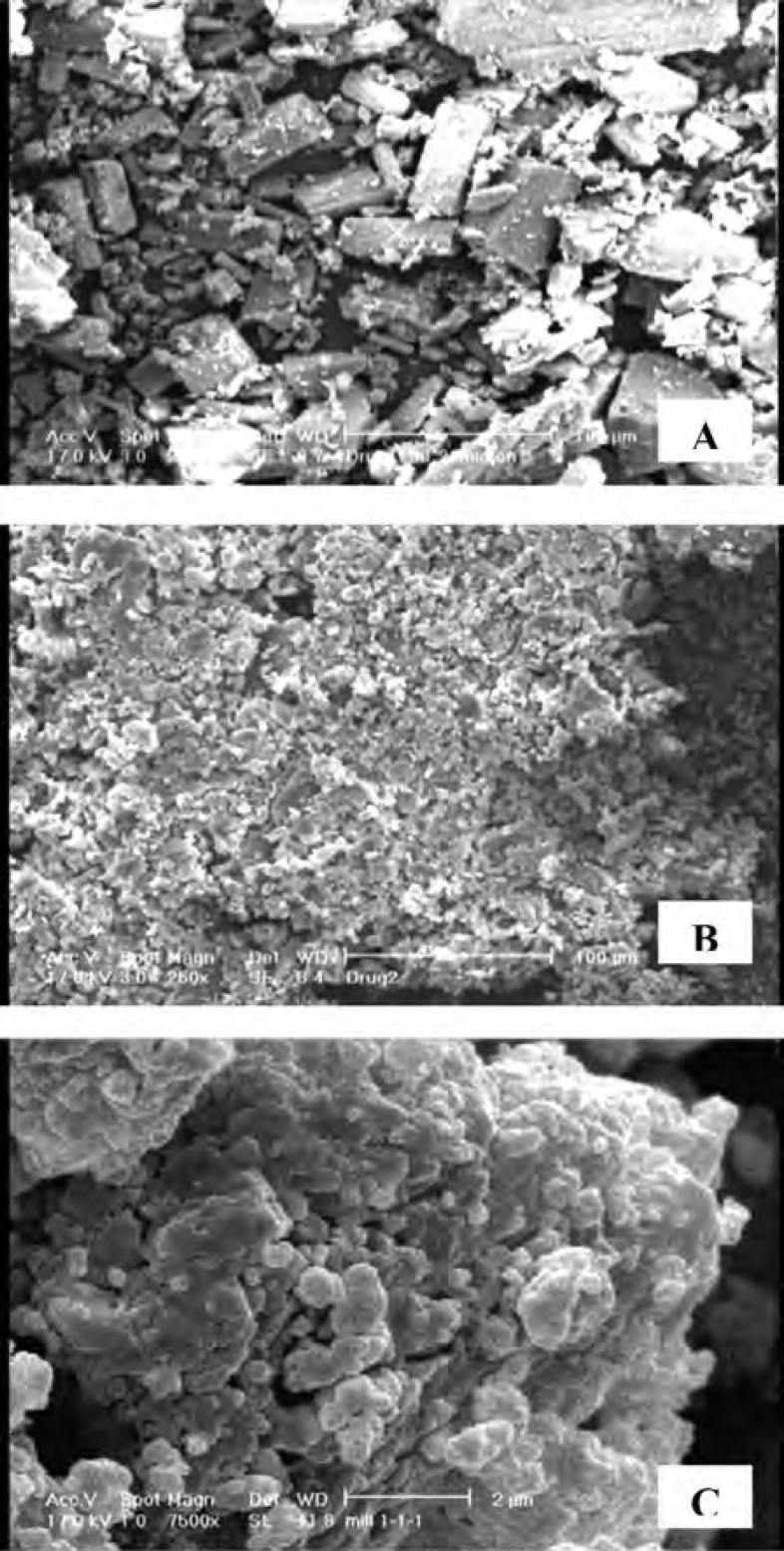
Scanning electron micrographs of: A) untreated CLA (× 250), B) co-ground sample (S6) (× 250) and C) co-ground sample (S6) (× 7500).

Figure 6 shows the scanning electron micrographs of untreated drug as well as co-ground formulation consisted of drug: SLS : PVP ratio of 1:1:1 (S6) in which distinct differences in the morphology of samples could be observed. The micrographs show columnar-shaped crystals for untreated CLA, while the co-ground particles were irregular in shape and aggregated into small clusters. The existence of PVP in the formulation could be considered as the cause of particles aggregation during grinding process.

*Stability studies*

Ternary ground formulation (S6) was kept in accelerated stability conditions and then the physical properties of the mixture were studied. Based on Table 4, the CLA dissolution almost remained constant compared to the fresh sample, after exposing to the stability conditions for three months. There was not any significant difference between the DE_10_ values (p > 0.05), although a slight difference was observed in DE_30_. In addition, the drug assay in the co-ground mixture (95.54 ± 0.17 %) did not show any significant changes in the comparison with the fresh sample, which confirms that the formulation remains stable during the study.

The results obtained from particle size analysis (Table 4) revealed that even after exposing to the stability conditions, the mixture could form nanoparticles with d(0.9) equal to 521 nm after dispersing in water.

**Table 4:** Physicochemical characteristics of the co-ground mixture (S6) after conducting accelerated stability studies (45oC, 75 % RH) (n = 3, mean ± SD).

**The variable**	**Time (months)**	
	**0**	**3**
DE_10_ (%)	72.18 ± 1.018	72.33 ± 0.42
DE_30_ (%)	88.85 ± 0.846	84.95 ± 0.88
Drug assay (%)	96.21 ± 0.23	95.54 ± 0.17
d (0.5) (μm)	0.184	0.174
d (0.9) (μm)	0.531	0.521

## Conclusion

It can be concluded that CLA dissolution rate was improved significantly compared to the intact drug using ternary dry ground mixtures including PVP and SLS. CLA nanocrystals with enhanced saturated solubility were formed after dispersion of the co-ground mixture in water. Besides, the crystalline structure of the drug was not affected during the grinding process. Therefore, co-grinding method could be considered as a useful approach in the preparation of various rapidly dissolving formulations containing CLA. This could potentially lead to improved bioavailability of CLA dosage forms. 
